# HE-YOLOv5s: Efficient Road Defect Detection Network

**DOI:** 10.3390/e25091280

**Published:** 2023-08-31

**Authors:** Yonghao Liu, Minglei Duan, Guangen Ding, Hongwei Ding, Peng Hu, Hongzhi Zhao

**Affiliations:** 1 School of Information, Yunnan University, Kunming 650500, China; 2 Yunnan Province Highway Networking Charge Management Co., Kunming 650000, China; 3 Research and Development Department, Youbei Technology Co., Kunming 650000, China; 4 Key Laboratory of Anti-Jamming, University of Electronic Science and Technology, Chengdu 610000, China

**Keywords:** road defect detection, attention module, convolutional neural network, YOLOv5s, image processing

## Abstract

In recent years, the number of traffic accidents caused by road defects has increased dramatically all over the world, and the repair and prevention of road defects is an urgent task. Researchers in different countries have proposed many models to deal with this task, but most of them are either highly accurate and slow in detection, or the accuracy is low and the detection speed is high. The accuracy and speed have achieved good results, but the generalization of the model to other datasets is poor. Given this, this paper takes YOLOv5s as a benchmark model and proposes an optimization model to solve the problem of road defect detection. First, we significantly reduce the parameters of the model by pruning the model and removing unimportant modules, propose an improved Spatial Pyramid Pooling-Fast (SPPF) module to improve the feature signature fusion ability, and finally add an attention module to focus on the key information. The activation function, sampling method, and other strategies were also replaced in this study. The test results on the Global Road Damage Detection Challenge (GRDDC) dataset show that the FPS of our proposed model is not only faster than the baseline model but also improves the MAP by 2.08%, and the size of this model is also reduced by 6.07 M.

## 1. Introduction

The highway industry started in 1988, and is divided into five stages, which are the initial stage, rapid development stage, accelerated development stage, leapfrog development stage, comprehensive standardization and high quality development stage. With the development of society, the scale of China’s highways has jumped to being one of the world’s largest, and the highway mileage is the highest in the world. However, with the completion of the road and due to its use, because of the weather, vehicle weight extrusion, and other issues, the road will experience cracks, potholes, and a series of damages, which will greatly increase the probability of car accidents due to road defects.

According to the survey [1], although only about 1% of traffic accidents in China are caused by this factor of improper road quality. This does not indicate that traffic accidents are less associated with improper road quality, and unfavorable road conditions often induce and exacerbate traffic accidents. Abroad, 11,386 people lost their lives in India [2] during the four-year period from 2013 to 2016 alone due to traffic accidents caused by road defects, and this does not include minor accidents. In the United States, half of all traffic accidents are characterized by poorly maintained roads. According to the U.S. [3,4] Department of Transportation, more than 40,000 people lose their lives each year in the United States due to problems such as road defects and road icing.

At present, the development of road defect detection has broadly gone through three stages: traditional manual detection, semi-manual semi- automated detection, and fully automated detection [5,6]. Traditional manual detection methods, i.e., the walking human eye observation method, the sitting car video screen measurement and reading method, and the camera measurement method. These methods have great disadvantages, such as the road mileage being too large, resulting in its cost of human and material resources, manual detection has a complex human factor, which is not conducive to the objective assessment of pavement defects, and manual detection for the safety of road inspectors is also very unfavorable. Semi-artificial semi-automatic detection is mainly performed with drones equipped with high-resolution cameras to collect images, followed by human detection. In recent years, with the rapid development of several new technologies, such as computers, target detection, GPS, digital CCD, etc. [7,8,9], it is possible to automatically detect road defects. This type of detection has many advantages compared with the first two, such as carrying out real-time pavement damage image acquisition, the use of high-resolution digital image sensors, greatly improving pavement defects detection accuracy, integrated the GPS positioning function of the automatic detection system to achieve the precise location of pavement damage, for the road staff maintenance and management to provide a convenient fix, and finally by entering the target detection algorithm for the collected data. The detection of the collected data set is performed by inputting the target detection algorithm, which improves the detection efficiency.

Starting from the detection using the traditional ML model, Li et al. proposed a new unsupervised multiscale fusion defect detection algorithm (MFCD) [10], which improved the MAP by 22%, and recall by 12%, respectively, over the then state-of-the-art methods in the AigleRN, CRD, and APR datasets selected in the paper. In the same year, Ai et al. [11] proposed a support vector machine-based method to compute probability maps using multi-scale domain information and pixel intensity for this problem of road defect detection [12], which also improved a part of the performance on the CFD dataset. With the continuous development of society, computer vision based on deep learning has been widely used in our lives. More and more people have also started to engage in road defect detection by deep learning, and they have proposed various models for road defect detection, most of them rely on R-CNN, SSD, and YOLO as the main models for detection, and have achieved good results. Ronny et al., in a model with Resnet as the main backbone network, the GAPs in the public dataset reached 84% F1. Kasthurirangan Gopalakrishnan et al. [13], used the best weights obtained by training the ImageNet dataset with the VGG-16 model as pretraining weights and adapting this weight to the LTPP dataset using the idea of migration learning, and achieved the best performance compared to the model proposed at that time. Sukhad Anand et al. [14] proposed a model for real-time detection of road defects based on SqueezeNet backbone network and achieved a significant improvement in accuracy on the standard road defect datasets GAPS, ICIP. Liu et al. [15] proposed a two-step road defect detection and method based on convolutional neural networks, first by using an improved YOLOv3 model to detect the dataset and an improved U-Net to segment the cracks in the dataset for training, and finally achieved an F1 score of 90.58% on the CFD dataset. Pouria Asadi et al. [16] developed an efficient and beneficial platform that combines speed and detection performance, using RGB-D sensors and target detection models such as Faster-RCNN and SSD, achieved 97.6% performance for road defect detection on the PA VDSI2020 dataset.

The above-proposed model detects mainly in three ways. The first traditional way is to observe the defects only by naked eyes which is not only time-consuming and labor-intensive, but also poses a certain danger to human beings; the second semi-automatic way, although it makes up for the drawbacks of the previous way by some technical means, it is also helpless in the face of such a large mileage of highway. With the development of artificial intelligence, fully automated detection has been ushered in, which improves the efficiency of the detection to a certain extent by sending the captured dataset to the proposed optimization model for detection. However, it is well known that most of the current models in this class are based on two-stage detection models for detection. Although the success rate of this type of model is greatly improved, this type of model has many parameters that make its detection very slow. Therefore, in the face of such a large amount of data, in order to check and repair the defects of the pavement in time, we selected YOLOv5s, a single-stage detection model with excellent generalization, as the baseline model, which as the baseline model is not only effective in detecting most of the publicly available target detection datasets but also has a very fast detection speed. After selecting it as the baseline model, we tailor the model and add a replacement module based on it, and finally obtain an optimized model that can both guarantee the speed and improve the performance. The contributions of this paper can be summarized as follows:

(1) The feature fusion part was modified to obtain a feature map more suitable for this work and one detection head was reduced.
(2) The number of C3 modules of the backbone network was pruned.
(3) Introduced Ghostmodule and BottleneckTransformer modules in the SPPF module to obtain a new SPPF module.
(4) Added NAMAttention attention mechanism between the backbone and the Neck network.

## 2. Related Work

### 2.1. The YOLO Family of Models

In pursuit of real-time target detection, Joseph Redmon et al. proposed You Only Look Once [17], the pioneer of the YOLO series algorithm, in 2016. The detection speed of this model is quite fast, and it can quickly process 45 photos in one second, which is basically able to fulfill the requirement of real-time detection in that era. YOLOv1 proposed the idea of transforming target detection into a regression problem, using the whole picture as the input of the network, and after just one neural network, the position of the bounding box and its corresponding class can be obtained. However, the YOLOv1 model is not so useful for the detection of some objects in close proximity, and the performance is weaker for the detection of objects with poor angular position. So in the following year, the original authors proposed the YOLOv2 [18] model, based on the YOLOv1 model with a lot of improvements. YOLOv2 introduces the Anchor mechanism in the Faster-RCNN [19]. The suitable Anchor box is obtained by clustering the training set by the method of K-means clustering, which has a great improvement on this index of the recall rate. It also combines the fine-grained features of images and improves the detection performance of small targets by connecting deep features to shallow features. In 2018, the original authors summarized the innovations proposed in YOLOv2 and continued to apply the useful ones, based on which they proposed a new Darknet-53 network with a fusion of FPNs, and obtained the YOLOv3 [20] model by replacing Softmax as a classifier with logistic regression. In 2020, Alexey Bochkovskiy [21], in contact with Redmon, tested a full range of some strategies of deep learning on the basis of the YOLOv3 model and obtained strategies that are helpful to the model: WRC, CSP, CmBN, etc., which were added to the YOLOv3 model to satisfy the detection speed and performance of balanced development.

In the following years, researchers have proposed several improved models based on YOLOv4. Wang and Alexey et al., authors of YOLOv4, proposed ScaledYOLOv4 [22], a powerful model shrinkage method designed for small models to balance computational cost and storage bandwidth. The model can modify the depth, width, and resolution. The structure of the network can also be modified. CSP-ized YOLOv4, YOLOv4-tiny [23], and YOLOv4-large were proposed under this model, and Cai et al. proposed YOLOv4-5D [24] to apply it to autonomous driving technology, and this model has improved the detection performance on BDD and KITTI datasets compared to the baseline model YOLOv4. Shortly after YOLOv4 was released, Ultralytics released the first version of YOLOv5, which is comparable to YOLOv4 in performance and is loved for its fast inference speed. There are four versions of this model, and although the authors of this model did not write an official paper, a large number of papers have been proposed based on this model. Zhu et al. proposed TPH-YOLOv5 [25], the biggest strategy of this model is to integrate the TPH detection head into YOLOv5, and thus locate objects quickly in high-density road scenes. Yan et al. used YOLOv5 as a baseline model and proposed an improved YOLOv5s for a lightweight apple target detection method for picking robots, which achieves quite good metrics in terms of performance and speed. YOLOx [26] is a summary of all YOLO series target detection algorithms proposed by Kuangwei Technology in 2021. Ge et al., by combining experiments on each innovation point in the field of target detection in the next two years, finally concluded that the inclusion of innovations such as decoupling head, data enhancement, label assignment, and Anchor-free mechanism can maintain the efficient inference of YOLO series models while making substantial improvements to the performance.

### 2.2. SPP and Its Improvement

In order to solve the problem of trimming and scaling the playing process of images that makes them distorted, and to deal with repeated feature extraction by convolutional neural networks, He and their team proposed the spatial pyramidal pooling structure SPP [27], which greatly reduces the cost of computation and also accelerates the speed of generating candidate frames. Later, the author of YOLOv5, Glenn Jocher, modified the SPP by changing the pooling from CONV-BN-SILU operation on the original features to k = 5, 9, 13, respectively. Later, Mission proposed YOLOv6 [28], in which the SPPF was improved by changing the activation function SiLU in it to RELU activation function, making a single ConvBNReLU 18% faster than ConvBNSiLU. Inspired by SPP, Google’s team proposed the ASPP module in the DeepLabv2 [29] model, which processes each feature extracted at different sampling rates using parallel null convolution and fuses them to obtain the final features. Liu et al. introduced an improved Inception structure into the RFB module by simulating the human perceptual field [30], which substantially increased the perceptual field and thus improved the feature extraction capability of the backbone network. YOLOv7 [31] added a CSP structure to expand the perceptual field in spp, which has a large residual variation to assist optimization and feature extraction, and although its performance is good, its computational cost increases a lot. Therefore, it is especially important to propose an effective model to enlarge the perceptual field with a limited computational cost.

### 2.3. Feature Fusion

In target detection, we extract features by using different backbone networks with low-level features, mid-level features, and high-level features. Low-level features have a lot of detailed information, but their semantic nature is low making the extracted features contain more noise. On the contrary, high-level features have a lot of semantic information, but their low resolution makes them less capable of perceiving features in detail. Therefore, a fusion of low-level features with high-level features can take the essence and discard the dross. Features that contain both high semantic information and high detail information are obtained.

In view of this idea, He et al. proposed the FPN module [32] in Faster-RCNN for the first time, and accomplished multi-scale target detection by fusing high-level features with low-level features after an up-sampling operation. Later, Retinanet [33] network, YOLOv3, and SSD [34] extended FPN to perform the above operation on more features, and finally obtained multiple fused features. However, due to the more homogeneous way of FPN structure, in order to obtain more scale information, Liu proposed a two-way fusion model in Panet [35], whose general idea is also to introduce the inverse idea of FPN by performing down-sampling operations on low-level features and a fusion of high-level features. Later, considering the simplicity of the FPN network structure, an adaptive feature fusion approach ASFF [36] was proposed in YOLOv3, by filtering out the useless information from other layers of features and keeping the useful information. After experiments, it was found that this structure can effectively improve the detection performance and its generalization is good enough to be applied to most data sets. Zhou [37] proposed a bridging network to fuse multi-scale features to obtain more recognizable and scale-sensitive features, and fed them into a boundary-aware decoder network, which achieved very good results.

### 2.4. Model Pruning

When we train a redundant network model for inference, the limited computational resources can make us bottleneck in training the model.

Micro-YOLO [38] is a proposed pruning model based on YOLOv3-tiny. By replacing the convolutional layers in YOLOv3-tiny [39] network with lightweight convolutional layers DSConv and MBConv, and designing a progressive channel-level pruning algorithm to reduce the number of parameters and computational cost while maintaining the detection performance. SlimYOLOv3 [40] is also based on YOLOv3, which is first trained sparsely to obtain the scale factor of each channel, and then removes those channels with small scale factors, and the pruned model is further fine-tuned on the dataset to obtain the detection effect. Then the sparse training is continued until the pruned model meets the pruning rate requirement, which is considered to complete the experiment. The essential idea of this paper is that the channel scaling factor imposes L1 regularization and prunes the less informative feature channels to achieve channel-level sparsity in the conv layer. YOLOv4-tiny is a major pruning of the YOLOv4 model for the backbone network part, feature extraction part, and feature fusion part, respectively, and the less effective strategy proposed is YOLOv4. The YOLOv5-lite [41] backbone network is selected with a lighter Shuffle Block and the Focus layer is removed to reduce the number of slice operations and the number of times the C3 module is used. Finally, the detection head is pruned to obtain a lighter YOLOv5 model.

## 3. The Proposed Model

### 3.1. YOLOv5 Model

**Figure 1:**
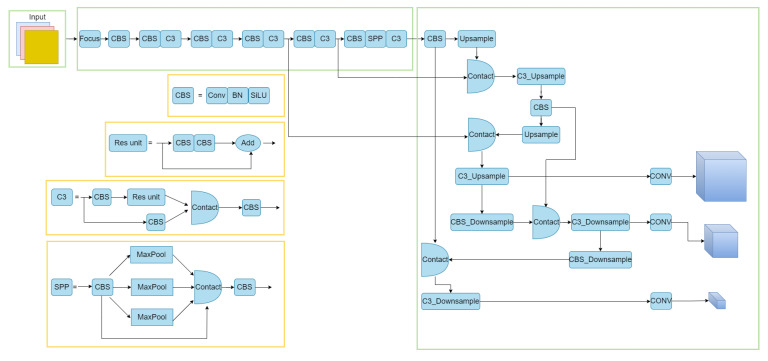
YOLOv5s model structure.

YOLOv5 is an algorithm obtained by adding some new and improved ideas based on YOLOv4, which is divided into YOLOv5s, YOLOv5m, YOLOv5l, and YOLOv5x. Due to the limited equipment in this work, and considering that the practical application of defect detection on roads is a time-consuming and labor-intensive task, this paper takes the lightweight model YOLOv5s as the baseline model, in the pursuit of detection performance while also meeting the efficiency requirements. As shown in Figure 1, YOLOv5s has three parts, the backbone network, the neck network, and the detection network. The backbone network is composed of the Focus module, CBS module, C3 module, and SPP module. The neck network is a feature fusion network obtained by combining the FPN network and PAnet, which achieves bi-directional fusion and provides both good detail information and a large amount of semantic information for subsequent detection work. The detection network is processed by a two-dimensional convolution of the fused features, and finally outputs 20 × 20, 40 × 40, 80 × 80 images for detection respectively.

### 3.2. Pruning of the Backbone and NECK Networks

**Figure 2:**
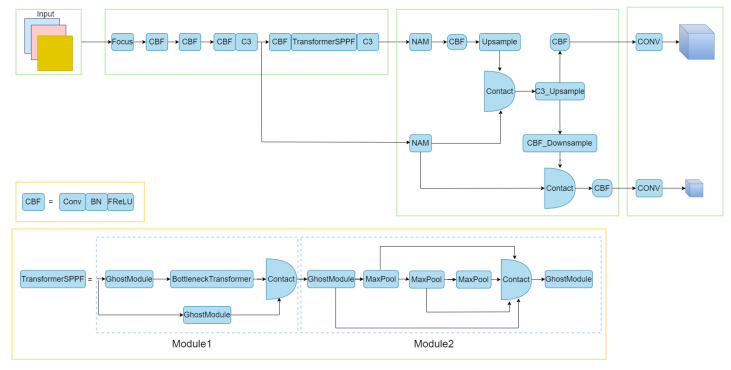
The figure shows the diagram of our improved HE-YOLOv5s model. By comparing Figure 1 and Figure 2, we can carefully find that our proposed model cuts some modules and replaces some modules, such as converting CBS to CBF, a process that replaces the SILU activation function with the FRELU activation function. Another improved SPPF is shown as TransformerSPPF in Figure 2, which is divided into two parts: module 1 and module 2. Module 1 is obtained by improving the BottleneckTransformer module in the second figure in Section 3.4, and module 2 is obtained by replacing the convolution module in the SPPF part with the Ghostmodule.

Considering that the road defect detection performed in this paper is a work for non-large target detection, a more concise and effective feature fusion network is obtained by pruning the NECK network part appropriately in this paper. As shown in Figure 2, this paper focuses on the 1024 × 1024 (feat2) size and 512 × 512 (feat1) size features extracted from the backbone network part, and the results obtained after this processing are similar to the feature fusion structure in Tiny-YOLOv4. The fused features are upsampled and downsampled, where the upsampled features are then used as the first detection head through a CBF operation and a one-dimensional convolution operation, and the downsampled features are used as the first detection head through a CBF operation and a one-dimensional convolution operation. The feature maps of the downsampling operation are fused with the feat1 features after upsampling and downsampling, and then fused with the CBF operation and 1D convolution operation as the second detection head.

After the modification of the Neck network, as this paper mainly focuses on the processing of feature maps of 1024 × 1024 and 512 × 512 size, the C3 module is removed from the backbone network to complete the pruning of the backbone network under the premise of not reducing the performance too much.

### 3.3. Activation Function

**Figure 3:**
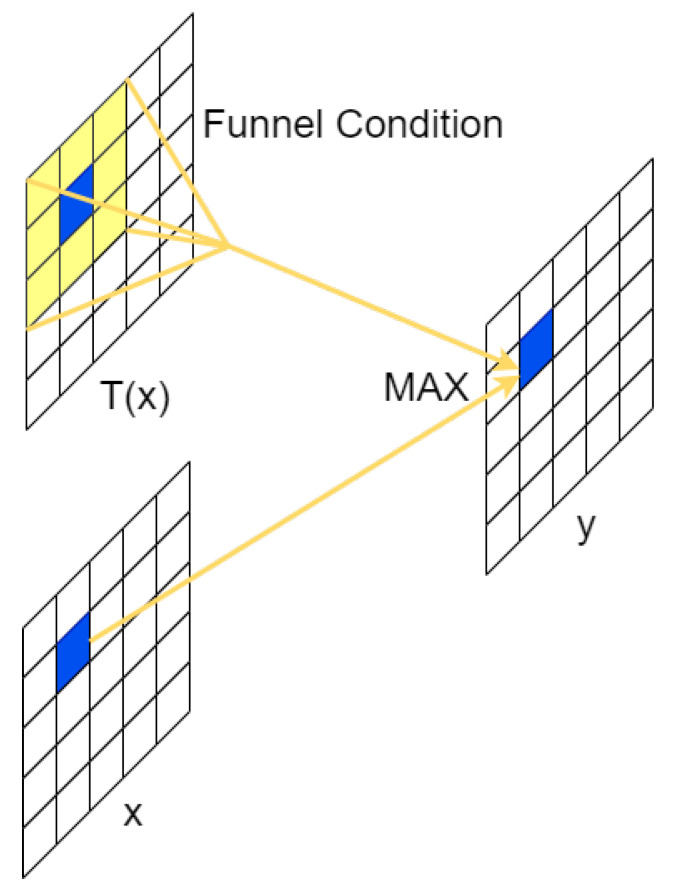
Frelu activation function structure diagram.

FRULU function [42] is an activation function that can achieve pixel-level spatial information modeling capability proposed by Kuang Shi Technology and Hong Kong Polytechnic University in 2020, and FRELU is obtained by extending RELU and PRELU to 2D activation functions after adding negligible computational cost. As shown in Figure 3, this activation function is the conditional part of the max function by deep separable convolution. This function is converted into a 2D funnel condition, which solves the spatial insensitivity problem in the activation function and also makes the regular convolution have the ability to capture the complex visual layout.

In this experiment, after replacing the SILU activation function and ReLU activation function in the convolution and GhostModule [43] with FReLU activation function, we found a certain performance improvement with a small increase in computational cost after the experiment. This is because FRELU is capable of capturing irregular and detailed spatial information, and the defect detection for roads in our present work happens to fall under this feature, so the activation function is able to capture the context better, which helps a lot in understanding small targets and irregular objects.

### 3.4. BottleneckTransformer-SPPF Module

In this section, we present the BottleneckTransformerSPPF [44] module. As shown in Figure 2, this module combines module 1 and module 2.

**Figure 4:**
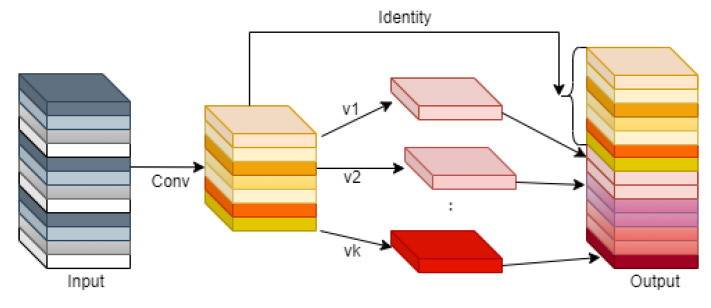
Structural diagram of Ghostmodule.

**Figure 5:**
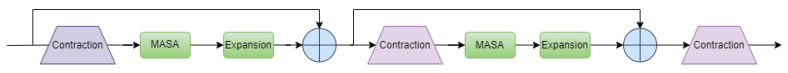
Structural diagram of Bottleneck Transformer. This module actually replaces the 3 × 3 null convolution module in the Resnet50 Bottleneck module with the Multi-Head Self-Attention (MHSA) [45].

Module 1 is modeled after the C3 module in the YOLOv5s model, as shown in Figure 1. We replaced the convolution in the C3 module with the Ghostmodule and introduced the BottleneckTransformer module. In this process, as shown in Figure 4, the Ghostmodule module we used generates part of the real feature maps by using the normal convolution, and then uses these real feature maps to obtain the phantom feature layer by linear transformation, and finally combines the two to obtain the complete feature layer. This makes the feature information we obtain more complete. We introduce the BottleneckTransformer module, as shown in Figure 5, which is a conceptually simple but powerful backbone architecture, combining self-attention with computer vision. By replacing the three bottleneck blocks of null convolution in the ResNet network with global self-attention, it not only reduces the parameters, but also improves the performance of small targets very significantly.

In module 2, we are processing the SPPF module, the SPPF module is improved on the basis of the SPP obtained. As shown in Figure 1, the SPP module is actually the input obtained first directly to the output as the first branch, and then the input was pooled kernel for 5 × 5, 9 × 9, 13 × 13 pooling to obtain the second, third and fourth branches, and finally these four branches Finally, these four branches are fused to obtain the output processed by SPP. SPPF is a simplified process of SPP, that is, the original input is not directly after convolutional processing as the first branch, and then after convolutional processing of the input through the pooling kernel 5 × 5 pooling to obtain the second branch, so that in turn 9 × 9 pooling, 13 × 13 pooling to obtain the third branch and the fourth branch will eventually be fused after convolutional processing to obtain the complete output. We replace the convolution module in the SPPF module with Ghostmodule to obtain more complete information, which not only greatly reduces the number of parameters, but also obtains more perfect features.

### 3.5. Replacing Upsampling

**Figure 6:**
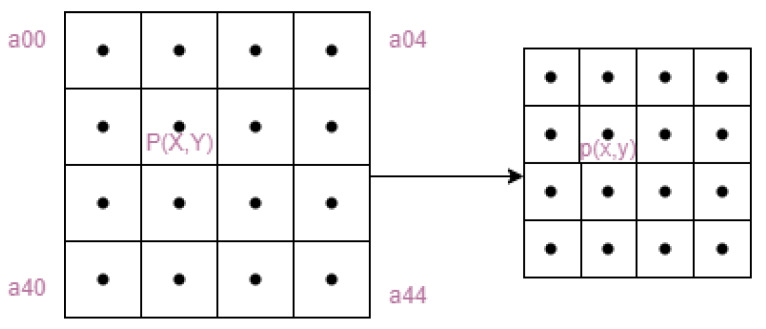
Nearest-neighbor interpolation schematic.

In deep learning, we use a convolutional neural network to extract features from the input image, which then reduces the size of the image, and we restore the image to a certain size to fuse it with other features. This process is generally known as resolution change, or upsampling. There are three main types of upsampling: interpolation, deep learning, and maximum pooling. In the YOLOv5 model shown in the figure, we up-sample feat2 by convolving it to match the size of feat1 for feature fusion. In the original YOLOv5 model, the authors used nearest-neighbor interpolation. As shown in Figure 6, by inserting two unknown values P1 and P2 into the a-plot, P1 = 0 since the distance of P1 from a 0 grey value pixel is less than the distance of 100 grey values. similarly, P2 = 100.

**Figure 7:**
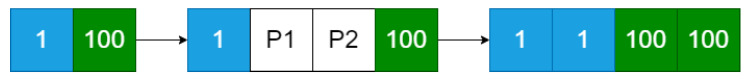
Bicubic interpolation schematic.

The computational cost of nearest neighbor interpolation is small, but the processing of images using this method can lead to jagged images. This paper, therefore, introduces bicubic interpolation without adding too much to the computational cost of the calculation. As shown in Figure 7, the bicubic interpolation algorithm calculates the weights based on the 16 surrounding pixel points and accumulates them to obtain the pixel values of the added points. Among other things, the method is based on the following idea:

(1) First, an image of size m × n is scaled k times to obtain an image of m/K × n/K.
(2) Since the pixel points in the original image are known, we need to obtain the pixel values of the scaled image, we must find the pixel (x,y) corresponding to each pixel point (x,y) of the scaled image in the original image.
(3) The 16 nearest pixel points of the original image from pixel (x,y) are compared with pixel (x,y), and the resulting parameter is used as the nearest 16 pixel points for calculating (x,y).
(4) Find the 16 pixel point weights by the BiCubic function, where the BiCubic function is shown in Equation (1), and finally add the 16 pixel point weights to obtain the pixel value (x,y) after the scaled image, and finally the corresponding coordinates (x,y) of the original image can be obtained according to the proportional conversion relationship.

(1) $$W\left(x\right)=\left\{\begin{array}{c} \left(a+2\right){\left|x\right|}^{3}-\left(a+3\right){\left|x\right|}^{2}+1,\;\;\;for\;\;\left|x\right|\leq 1\\ a{\left|x\right|}^{3}-5a{\left|x\right|}^{2}+8a\left|x\right|-4a,\;\;\;for\;\;1<\left|x\right|<2\\ 0,\;\;\;otherwise \end{array}\right.\;$$

where, a=−0.5, *x* represents the horizontal coordinate of the original image, W(x) denotes the weight of each pixel point calculated.

### 3.6. Adding Attention Mechanism

In order to apply more computational resources to important tasks, in this paper, after trying to add various attention mechanisms [46,47,48,49,50,51,52,53], modules after the Feat1, and Feat2 feature maps, we found that the NAMAttention module for this work is a module with improved performance.

**Figure 8:**
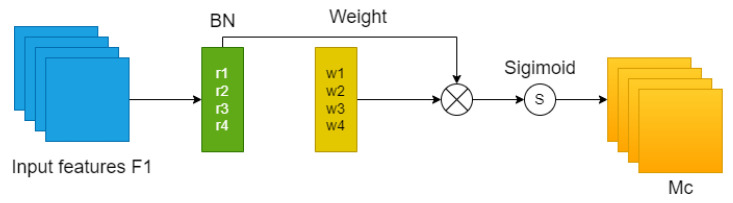
Channel attention mechanism.

**Figure 9:**
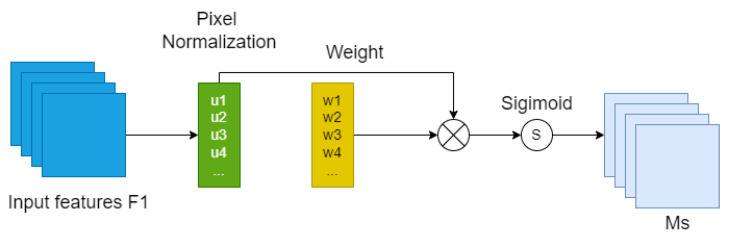
Spatial attention mechanism.

As shown in Figure 8, this module uses the scaling in BN directly to calculate the attention weights without additional calculations and parameters, such as additional fully connected layers and convolution, and suppresses insignificant features by adding regularization. This module is similar to the CBAM module divided into two parts: as shown in the figure, the channel attention module is a normalization operation for the input features at different scales, which uses the scaling factor in BN, i.e., Equation (2), and then the corresponding weights are obtained after the Wr operation in Equation (3), and finally the output features are obtained by the Sigmod function. The only difference between the spatial attention module and the channel attention module is the normalization operation; in the spatial attention module, as shown in Figure 9, we perform pixel normalization on the input features, and finally perform the same operation as the channel attention module to obtain the final output features, i.e., Equation (4). In addition, we add a regularization term to the loss function, i.e., Equation (5), to suppress the unimportant features to rationalize the use of resources.

(2) $$B_{out=}BN\left(B_{in}\right)=\gamma \frac{B_{in}-\mu_{\beta }}{\sqrt{\sigma_{\beta }^{2}+\varepsilon }}+\beta$$

(3) $$M_{c}=sigmoid\left(W_{\gamma }\left(BN\left(F1\right)\right)\right)$$

(4) $$M_{s}=sigmoid\left(W_{\gamma }\left(BN_{s}\left(F2\right)\right)\right)$$

(5) $$Loss=\sum_{\left(x,y\right)}l(f(x,W),y)+p\sum g(\gamma )+p\sum g(\lambda )$$

## 4. Experimental Details and Discussion

### 4.1. Experimental Details

#### 4.1.1. Experimental Environment

This work was carried out in the laboratory on personal equipment for experimental training and performance evaluation, and quantitative analysis of the experimental results was carried out. This work was based on Pytorch to conduct the experiments and we configured the experimental environment via Anoconda. The hardware of the devices was CPU: R7-5700X; System: Windows; GPU: Nvidia 3060/12 gb, and cuda12.0, a version of cudnn8500, were used to conduct the experiments.

#### 4.1.2. Evaluation Metrics

In order to validate the effectiveness of the model proposed in this work when applied to road defect detection, the following metrics were introduced to evaluate the model.

mean Average Precision (mAP): The mAP refers to averaging the AP over all categories. the AP is called the mean accuracy, which is an average of the accuracies at different recall points.

Frames Per Second (FPS): FPS refers to how many frames per second, i.e., how many images can be processed by the target network we are using. In simple terms, it is how often the image is refreshed, and there are so many road defects all over the world that it is especially important to obtain a model that processes data relatively quickly.

Size: The size of a model is a clear indication of whether or not a model will maintain performance while keeping an eye on the computational cost of the model, which can help us reduce training time significantly.

In addition to the above two evaluation metrics, we have some other metrics to evaluate our proposed model, and here we introduce some concepts first. They are helpful in calculating the three evaluation metrics Precision, Recall, and F1-score.

TP: number of samples correctly predicted by the model to be in the positive category
FP: number of samples incorrectly predicted by the model to be in the positive category
FN: number of samples incorrectly predicted by the model to be in the negative category

Precision and Recall: precision and recall are two metrics widely used in the fields of information retrieval and statistical classification to evaluate the quality of results. Among them, the precision rate, also known as the finding rate, indicates the ratio of samples classified as positive categories that are truly positive. Recall, also known as the check all rate, indicates the proportion of samples that are truly positive categories that are correctly categorized as such. The formulas for both are shown in Equation (6) and Equation (7), respectively:

(6) precision=TP/TP+FP

(7) recall=TP/TP+FN

Whereas F1-score (F1) is a combination of precision and recall, it is the reconciled average of the two, which is calculated as shown in Equation (8):

(8) F1=2(precision×recall)/precision+recall

#### 4.1.3. Training Model

In this paper, the baseline model YOLOv5s was trained after dividing the training set, validation set, and test set. In the process of training the baseline, we did not select the weights of the Voc and Coco datasets for the YOLOv5s model training, but only let it load the backbone network for training, with a total of 300 Epochs trained, batchsize=16, and the learning rate was adjusted by cosine annealing method to adjust the learning rate. The best performing weights were used as the pretraining weights for the improved model, 30 epochs were frozen to find the optimal parameters, and the specific parameter settings were kept constant during this process.

#### 4.1.4. Dataset

**Figure 10:**
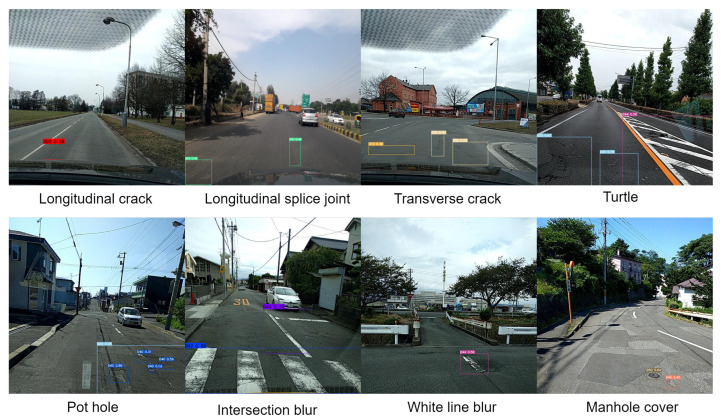
Selected data samples for eight road defect categories.

In this work, the dataset we use is the GRDDC2020 dataset [54], which is used for the Global Road Damage Detection Challenge. The dataset includes a total of 21,041 samples of road defects in three countries, namely Czechia, India and Japan, and the annotation file for the given test set is only officially available and we cannot obtain it. So in this paper, the GRDDC dataset was cleaned by removing the samples from the test set and removing the unlabelled samples from the training and validation sets to obtain 14,569 training samples. They were divided according to the ratio of 6:2:2 = training set:validation set:test set. We selected a total of 8 detection categories, namely longitudinal crack, longitudinal splice joint, transverse crack, turtle, pothole, intersection blur, white line blur, and manhole cover. Figure 10 shows data samples for the eight road defect categories we selected. For ease of operation in the experiment, the eight defect detection categories are noted as D00, D01, D10, D20, D40, D43, D44, and D50, respectively, and Table 1 shows the number of the eight defect detection categories.

### 4.2. Discussion

#### 4.2.1. Ablation Experiments

To demonstrate the feasibility of the model proposed in this paper, the experiments are conducted by reducing and adding modules to the baseline model step by step, as shown in (Table 2). First, a new feature fusion method suitable for target detection in this work dataset is proposed and one detection head is reduced to improve the training speed, because, in target detection work, it is not possible to improve the detection by fusing the feature maps several times performance, and we must find a feature fusion approach that is suitable for the dataset in a particular scenario. The new feature fusion approach proposed in this work reduces the unimportant structures in the model and greatly improves the speed with increased performance. Then we trimmed the C3 module of the backbone network to reduce its performance by 3.19% but increase its speed by about 30%. After that, by replacing the activation function, the performance was improved by 0.44% compared to the baseline model, and the speed was also improved by about 50%. Then we replaced the nearest-neighbor interpolation sampling with bicubic sampling in the neck network part, which slightly improved the detection performance with no speed loss. After that, we improved the SPP module by introducing the lightweight convolution module Ghostmodule as the convolution quickly, and added the Bottleneck Transformer module to improve performance by 1.02%. Finally, we added the attention mechanism NAM module to the feat1, and feat2 feature maps outputted by the backbone network, and the baseline model detection. In comparison, our proposed final model accomplishes a performance improvement of 2.06%, Fps improvement of 9.8, model size reduction of 7.07 M, and accuracy improvement of 1.01%.

#### 4.2.2. Comparison with YOLO Series Models

Since the model proposed in this work is not only improved in performance but also considerable in speed, in this section we compare the model proposed in this paper with the Tiny-YOLOv4, YOLOv4, YOLOv5s, and Tiny-YOLOx models, respectively. From the (Table 3), we can see that the improved YOLOv5s model is not only better than the other models in terms of performance, but also its model is the smallest. Although Tiny-YOLOv4 is faster than HE-YOLOv5s in terms of speed, its performance and accuracy are not as good as HE-YOLOv5s. We can also increase the speed while reducing the performance, but this will make our final application less effective than desired. Therefore, the HE-YOLOv5s proposed in this paper guarantees the performance while not falling behind in speed when performing the detection on the GRDDC2020 dataset.

#### 4.2.3. Compare My Optimization Model with Some Other Models

In this section, we perform a comparative analysis between our model and the best models from the GRDDC2020 competition, which are the same models that are trained with our selected training set, and then the best weights of the training are used to test them against the test set provided by the competition, which is evaluated by the F1-score score. We trained and tested the original training set with a 6:2:2 division (we did not have access to the test set of the contest), so this comparative analysis is informative. We obtained the F1-score by using our optimization model to train the dataset and computing the F1-score on the divided test set, where the results are shown in Table 4. By observing the results, we found that our score is completely better than the other models except for some differences with the first-place result. Of these models, most of them are trained based on single-stage detector YOLO and two-stage detector Faster-RCNN, and the speed of our model is obviously much faster compared to these two types of detectors. First of all, our proposed detector is a two-stage detector and the model is obtained with YOLOv5s (the lightest model among the various YOLOv5 models) as the baseline model, and each series of updates to the YOLO family of models is accompanied by an increase in speed and performance. Our proposed model is superior in targeting the F1-score, a metric that has some gap compared to the first place, but the speed is superior. Therefore, this is enough to prove the feasibility of our proposed optimized model.

#### 4.2.4. Comparison with Other Series Models

In this section, we have used the MobilenetV2-SSD, Efficientdet, Resnet50-Centernet, and Tiny-YOLOx models for the GRDDC2020 dataset, and the results are shown in Table 5 below. From Table 5, we can obviously find that in our model, compared to these excellent models, MAP and Precision have reached the first ranking, FPS has reached the second, and the parameters of the model are the third. In the combination of these evaluation indexes, our proposed model is superior to these classic models, but its MAP does not reach 100%, which means that we will miss the detection of some of the defects of the road. We still need to continue to search for the optimal model, in the pursuit of speed at the same time as improving the MAP.

## 5. Conclusions

In this paper, we use the YOLOv5s model as the basis and add the Transformer, NAM module, and some empirical techniques. Our experimental results show that HE-YOLOv5s achieves good performance on the GRDDC2020 road defect dataset. Therefore, if our proposed model is applied to detect road defects in every country in the world, the road defects be checked in advance in a very short time and the corresponding defects be repaired, which will reduce the number of traffic accidents in automobiles due to road defects, which is of great help to human safety.

Although our model achieves good results on the GRDDC2020 dataset, its detection MAP is only 58.87%, which means that there are some road defects that we cannot detect. Therefore, in the future, we will continue our research on this problem of road defects and work on proposing higher-performance detection models for the benefit of humankind. We will strive to leave no room for road defects to escape notice.

## Figures and Tables

**Table 1:** Details of the GDRRC2020 dataset detection.

Type and Number of Tests Performed	D00	D01	D10	D20	D40	D43	D44	D50
Numbers	5307	141	3501	6717	4523	639	4025	2888

**Table 2:** The result of the ablation experiment. The ‘-’ sign indicates that the operation corresponding to the first column of the table was not performed in the ablation experiment, and the ‘+’ sign indicates that the operation corresponding to the first column of the table was performed in the ablation experiment.

YOLOv5s HE-YOLOv5s							
Feature fusion	-	+	+	+	+	+	+
Delete parameter	-	-	+	+	+	+	+
SiLU to FreLU	-	-	-	+	+	+	+
BottleneckTransformer-SPPF	-	-	-	-	+	+	+
Add NAM	-	-	-	-	-	+	+
Map (%)	56.79	56.82	52.63	57.23	57.57	58.59	58.87
FPS (ms)	81.81	110.96	141.41	120.89	119.73	95.88	90.61
Size (M)	27.88	21.55	20.82	21.20	21.20	21.79	21.81
Precision (%)	81.01	76.55	76.10	78.73	80.39	81.23	82.02

**Table 3:** Comparison with YOLO series models.

	Tiny-YOLOv4	YOLOv4	YOLOv5s	Tiny-YOLOx	HE-YOLOv5s
Map (%)	52.45	55.67	56.79	58.70	58.87
FPS (ms)	160.22	41.14	81.81	72.90	90.61
Size (M)	23.08	250.41	27.88	19.91	21.81
Precision (%)	67.41	73.67	81.01	75.62	82.02

**Table 4:** Compare my optimization model with some other models (compare based on F1-score as evaluation metric).

	FR-CNN	YOLOv4	Ultralytics-YOLO	Improved YOLOv4	HE-YOLOv5s
Proposed team name	E-LAB	AIRS-CSR	IMSC	SIS Lab	Ynu
F1	0.47	0.55	0.67	0.63	0.65

**Table 5:** Comparison with other series models.

	Mobilnetv2-SSD	Effcientdet	Resnet50-Centernet	HE-YOLOv5s
Map (%)	45.94	40.05	56.69	58.87
FPS (ms)	96.97	28.65	60.15	90.61
Size (M)	18.29	15.45	127.94	21.81
Precision (%)	81.50	80.60	79.29	82.02

## Data Availability

No new data were created or analyzed in this study. Data sharing is not applicable to this article.
